# Supplementary material for: “…pretty much all white, and most of them are psychiatrists and men”: Mixed-methods analysis of influence and challenges in global mental health
Source: PLOS Glob Public Health. 2025 Mar 5;5(3):e0003923. doi: 10.1371/journal.pgph.0003923 (PMC11882038; doi:10.1371/journal.pgph.0003923)
Supplement: Attachment — Submitted filename: Response to Reviewers.docx [file pgph.0003923.s001.docx]

Dear Editors,

Thank you for taking the time to review our manuscript titled *“…pretty much all white, and most of them are psychiatrists and men”: mixed-methods analysis of influence and challenges in global mental health* [PGPH-D-24-01857]. We are grateful that our manuscript is being considered for potential publication in PLOS Global Public Health and for the reviewer comments that have strengthened our manuscript. Please find detailed responses to each reviewer comment below, with relevant pages in the marked manuscript in parentheses for ease of reference. We trust we have sufficiently addressed the concerns raised and look forward to your response.

Sincerely,
Study authors

**Editor comments**

**E1.1: Your current Financial Disclosure does not have Grant Recipient. However, your funding information on the submission form indicates a Grant Recipient "Dr Farah Shiraz". Please indicate by return email the full and correct funding information for your study and confirm the order in which funding contributions should appear. Please be sure to indicate whether the funders played any role in the study design, data collection and analysis, decision to publish, or preparation of the manuscript.**

Response E1.1: We corrected this as: “The Singapore Population Health Research Impact Centre (SPHERiC) provided funding for data collection and analysis (reference 026), through an award to Drs Shiraz and Howard…”

**E1.2: We note that your Data Availability Statement is currently as follows: "All relevant data are presented in this paper." Please confirm at this time whether or not your submission contains all raw data required to replicate the results of your study. Authors must share the “minimal data set” for their submission. PLOS defines the minimal data set to consist of the data required to replicate all study findings reported in the article, as well as related metadata and methods (**[**https://journals.plos.org/plosone/s/data-availability#loc-minimal-data-set-definition**](https://ddec1-0-en-ctp.trendmicro.com:443/wis/clicktime/v1/query?url=https%3a%2f%2fjournals.plos.org%2fplosone%2fs%2fdata%2davailability%23loc%2dminimal%2ddata%2dset%2ddefinition&umid=858884cc-86a8-42f8-acff-b6f297e0bea6&auth=8d3ccd473d52f326e51c0f75cb32c9541898e5d5-7e03f261fc90f21276298bf162486049afc6f054)**).**

Response E1.2: We clarified this as: “We are making the minimal data set publicly available through the LSHTM research repository.”

**E1.3: Please upload a copy of Figure 1a and 1b which you refer to in your text on page 6 and 22. Or, if the figure is no longer to be included as part of the submission please remove all reference to it within the text. Please provide separate figure files in .tif or .eps format.**

Response E1.3: We have uploaded these as separate files.

**Reviewer 1 Comments**

**Comment 1.1: This was an interesting paper in its attempt to answer some broad scoped questions. While the authors presented complex findings a coherent and methodical manner, with an awareness of the limitations of the sample itself, there are some missed opportunities to interrogate more comprehensively the results that are presented.**

Comment 1.1: Thank you for your praise and the time you have taken to consider our manuscript. We have addressed your subsequent comments and hope that our responses answer your concerns.

**Comment 1.2: A few general suggestions that would help improve the critical assessment of the results in the discussion would be as follows:**

**The paper goes to some lengths to differentiate to differentiate the field of “global” mental health as incorporating for eg social determinants of mental health more centrally than global health as a field. It feels like an arbitrary distinction given that the authors then throw in concepts of “public health” alongside “colonial” constructs of health. It will help to discuss this more critically – as currently it doesn’t really make it clear where and if the distinction lies, what the difference and value would be in conceptualising the field.**

Response 1.2: Thank you for acknowledging this. We agree that could be clearer and have revised - using a historical and critical contextualisation as: “*Historically, addressing the mental health of populations focused on investigating severe mental disorders in Europe and North America, with some cross-cultural explorations but limited “global” thinking [**3**]. Separate but related developments in academia and practice converged to inform the beginnings of the contemporary field of global mental health. Some of these developments included growing evidence of systematic injustices experienced by people with mental health conditions across the world and studies that demonstrated a range of effective treatments for mental disorders in low and middle-income countries (LMICs) [**3**]. Global mental health can thus be considered a sub-field of global health involving consolidation of the efforts of mental health actors across international borders, sectors, and professional specialties to improve the mental health of populations globally [...]. Against a backdrop of competing global health priorities, there is an urgent need to identify influential actors in this nascent field, to determine challenges, and to ascertain possible directions for its future development”* (p3).

**Comment 1.3: Similarly to above the idea that the field of “psychiatry” being equated with a biomedical model of mental health management feeds into an archaic (and false) narrative that the psychiatrists are only representative of a Biologically focussed approach. It will be helpful to give the reader a sense that this is not necessarily the case, and that perhaps the academic and non-clinical weight of those considered influential are not necessarily the norm. (The field of psychiatry itself has many subspecialties which are not biologically focussed at all) – and contributes to the stigmatization of mental health conceptualization. In fact, in the majority world there is more of a collaborative approach to mental health management policy and advocacy alongside psychiatrists/allied health and lived experience patient researchers. The paper will benefit from a more inclusive and thoughtful perspective of this.**

Response 1.3: Thank you and we agree that this could be more nuanced. As such, we added to the Discussion section: “*Despite evidence in the extant literature demonstrating the range of psychiatric approaches to mental health beyond those informed by the biomedical model, these alternatives may not be highlighted in mental health research, planning and practice. This gap between knowledge and practice was reflected in interviewee experiences. Considered alongside the historical roots of the global mental health field, critical engagement of and within the field is needed […]. Critical perspectives, from disciplines such as anthropology and sociology, could provide constructive avenues to renew ways of conducting knowledge generation and transfer to policy and practice to improve mental health outcomes*” (p 14). This interpretation is from current literature, cited on page 14, and reported experiences of interviewees who highlighted the dominance of the biomedical approach and biomedically focused psychiatry.

**Comment 1.4: The discussion would benefit from a bolder and more nuanced critique of the responses – acknowledging the narrow representation of the participants as has been highlighted. How has this centered the need/urgency for more specific focussed approaches rather than the general suggestions given in the discussion/concluding paragraphs?**

Response 1.4: Thank you for this suggestion. We revised the Discussion section and tried to address this as much as possible (pp 14-16). We also added the following in Limitations: “*Acknowledging that the representation of our study participants is limited despite our efforts in conducting purposive sampling was an important self-reflexive step for us as scholars grounded in adopting critical approaches and further research is warranted to address this […]. Third, our study was conceived broadly to ask global mental health actors who they considered to be influential in global mental health and about their experiences working within the field. Even though our representation of global mental health actors is limited, we obtained responses from participants who worked across many settings. As such, we have refrained from providing very specific suggestions and recommend further research to inform country-specific or community-specific solutions or to guide supranational efforts*” (p 16).

**Comment 1.5. As the authors introduce the impact and influence of COVID-19 pandemic early on in the paper as their point of departure, it was expected that there would be a more uniform thread on how this influenced the perception of who the influencers were? And the disparity highlighted between majority world and the the rest in terms of access, and response to the pandemic that invariably should have but didn’t quite center the value of the non-Western world in contributing to influence is an opportunity that the authors should explore and discuss in this paper to critically bring it all together.**

Response: 1.5: Thank you. first, we also anticipated a more uniform thread in our data but found this not to be the case. We have thus reframed our point of departure to take a longer-term perspective and reduce emphasis on implications of the COVID-19 pandemic while still noting that the pandemic had considerable consequences for the mental health and wellbeing of populations and data were collected at the height of the pandemic (p 2). Second, we agree there is value in highlighting the contributions that non-Western countries made to global mental health during the pandemic but this did not come out in our data so we did not add to this.

**Reviewer 2 Comments**

**Comment 2.1: My main problem with the draft is that there is an incongruence between the background (lack of relevance of global mental health despite its importance), the methods (identification of the most influential mental health actors and institutions through interviews and social network analysis), and the results (concentrated influence, lack of diversity). The piece needs a profound reorganisation to make the argument cohesive. As far as I see, the main contribution of the piece is to highlight the lack of diversity and representation in global mental health. However, if that's the main contribution, then the introduction needs to introduce that problem. At the moment, the introduction is very generic; it repeats what's already being said about the importance of mental health globally, but it doesn’t touch on the growing literature and discussion about diversity and representation in global health and global mental health. Focusing the entire piece on diversity and representation would better align the different sections of the piece.**

Response 2.1: Thank you for your useful comment. To address this we revised the Introduction section to remove focus on COVID-19 and instead focused on expanding the historical and critical approach we have taken to frame the study, which allows space for analysis of diversity and representation (p 2). We then mapped these questions to study objectives to ensure coherence between how the study is framed and Results and Discussion sections, and revised the Discussion section to align with this (pp 13-16).

**Comment 2.2: The first paragraph, before any context or results are given, is already making proposals about what should be done with global mental health. This section should only provide the context/situation, and proposals should be given once the findings are presented.**

Response 2.2: Thank you. We have revised the Introduction section so context is clearer (please see Responses 1.1 and 2.1).

**Comment 2.3: The second paragraph gives an intriguing rationale for the paper: "Against a backdrop of competing global health priorities and the “partial and temporary” nature of the work striving for universality within the field, there is a longstanding need to identify influential global mental health actors and determine challenges and gaps [4]. Doing so enables further areas of exploration and development to be delineated to help the field advance and attract greater prioritisation and resources." An immediate question is why?. In what sense does the identification of influential actors responds or solves the lack of priority and fragmentary nature of global mental health? It might do, but the article doesn’t explain this, and it is crucial.**

Response 2.3: Thank you. Please also see Responses 1.1, 1.2, 1.5, and 2.2.

**Comment 2.4: The section called “thematic analysis of interview data” points to three themes created through inductive analysis: (i) overly concentrated influence; (ii) insufficient global collaboration; (iii) lessons for broadening involvement. This is methodologically clear and solid and reveals what the paper is really about. Again, this is why the introduction needs to change.**

Response 2.4: Thank you for highlighting this. We agree that the Introduction insufficiently aligned with our topic and have revised this as you suggested (p 2). We trust that our efforts in re-contextualising our study and findings will make this clearer.

**Comment 2.5: Most quotations from interviews are interesting and rich. However, some require more explanation. For instance, Page 10 includes one that mentions the need to include people with lived experience at different levels, but there’s no mention of this in the analysis. Another quotation whose content is not contextualised or adequately used is the last one on page 13, where the dominance of English is mentioned by an interviewee.**

Response 2.5: Thank you for noting these gaps. To clarify the first instance, we added a paragraph in the Discussion section expanding on the potential role for people with lived experience and service users can contribute to the field of global mental health: “*As many interviewees highlighted, actively involving people with lived experience, by incorporating their perspectives into planning and implementation can complement existing ways of working within global mental health. However, associated challenges require that this is done with due care to mitigate the risks of misinterpretation and exploitation or further traumatisation of people with lived experience, and ensure adequate training and preparation of lay people to productively engage in professional mental health settings at the global level*” (p 14)

For the second instance, we appended a paragraph before the quote to contextualise it and illustrate a key challenge: “*Sharing experiences of living with and managing mental health conditions is typically expressed in ways that are most familiar to users. Although the unique voices of people with lived experience can highlight experiential realities and counter negative stereotypes, these perspectives may remain within certain social circles. This is especially true on social media platforms that allow for posting and sharing of user-generated content. For example, language can be either bridge or barrier when users post about their experiences as people primarily access and read content in languages they understand*” (p 13).

**Comment 2.6: The discussion section is probably the weakest at present. For the most part, it consists of generic, well-established, and often repeated recommendations for the development of public mental health systems in low- and middle-income countries (LMICs). These are only tangentially informed by the qualitative findings. The discussion should stem directly from the findings. There are points made in the discussion that are not sustained by the findings and where no justification is given. For example: “Despite potential difficulties, it is through greater representation of diverse voices that stigmatising mental health discourses will change.” The piece calls for unity and supranational coordination on the one hand, and local adaptability, diversification of voices, and inclusion of subnational and non-mainstream perspectives on the other. There’s a degree of contradiction or tension between these two calls that the piece doesn’t unpack or develop. Exploring this tension and its implications would make the piece stronger.**

Response 2.6: Thank you for this helpful feedback. We have significantly revised our Discussion section to focus on insights from our data (please see Response 1.4). To address the tension between the call for unity and coordination at the supranational level and the call for local adaptability, we posit that these two calls do not necessarily contradict each other. We partially clarified this as: “*As global mental health is conceived and practised across a wide range of populations, context-specific approaches to mental health must be supported. Fields such as cross-cultural psychiatry or ethnopsychology highlight the cultural variability of symptoms previously thought to be universally expressed and emphasise the importance of mental health research to be contextually attuned […]. Interviewees underscored the vital role that community-based organisations play in needs assessment and ensuring the feasibility and sustainability of mental health initiatives. Additionally, onus should be on national governments, where possible, to coordinate between international bodies and those at subnational levels. Many noted the success of policies and interventions was influenced by political championing and governmental commitment*” (p 15).
