# Supplementary material for: “…pretty much all white, and most of them are psychiatrists and men”: Mixed-methods analysis of influence and challenges in global mental health
Source: PLOS Glob Public Health. 2025 Mar 5;5(3):e0003923. doi: 10.1371/journal.pgph.0003923 (PMC11882038; doi:10.1371/journal.pgph.0003923)
Supplement: Attachment — Submitted filename: plos_AL.docx [file pgph.0003923.s002.docx]

The re-submitted version is an improvement from the original and attempts to be more cohesive. The presentation now of the results and the interrogation thereafter is more acceptable.

It still do feel it lacks some integration of the complexity of the subject of global mental health, and it currently reads as if there are separate and distinct “actors” – a word used frequently by the authors, that contribute, where as understanding global mental health often requires an understanding that the contributors are less clearly delineated and have more of a blurred spectrum of challenges/risks/support than clear separation of contributing factors. This is a personal reflection, and critique and may not necessarily need a full overhaul if Reviewer 2 is not in agreement.

The main comment I have is on is the COVID-19 inference. As mentioned in the original review – there was an expectation that seeing as this study data was collected during the pandemic, there would be some reflection on this? It is such an integral flash point in the understanding of the disparities in the global health space, and mental health itself was detrimentally affected. The paper currently doesn’t address the original comment/suggestion on how to integrate the contribution of COVID 19, but especially since the authors mention it – there must be some expansion and critique on this.

My original comment is as reference.

Additionally the authors now only mention that the data was collected in COVID in the last paragraph of the introduction, and they also mention only once in the results a comment by a participant.

If there is an intention to include a COVID reference then there must be some introductory thoughts around it, and more focus on its contribution in the discussion.
