# Supplementary material for: “…pretty much all white, and most of them are psychiatrists and men”: Mixed-methods analysis of influence and challenges in global mental health
Source: PLOS Glob Public Health. 2025 Mar 5;5(3):e0003923. doi: 10.1371/journal.pgph.0003923 (PMC11882038; doi:10.1371/journal.pgph.0003923)
Supplement: Attachment — Submitted filename: Response_to_Reviewers_auresp_2.docx [file pgph.0003923.s003.docx]

Dear Editors,

Thank you for the detailed second review of our manuscript titled *“…pretty much all white, and most of them are psychiatrists and men”: mixed-methods analysis of influence and challenges in global mental health* [PGPH-D-24-01857]. We are grateful for the additional comments that will allow it to be considered for publication in PLOS Global Public Health. Please find detailed responses to each reviewer comment below, with relevant pages in the marked manuscript in parentheses for ease of reference. We trust we have sufficiently addressed the concerns raised and look forward to your response.

Sincerely,
Study authors

**Reviewer 1 Comments**

**R1.1: The main comment I have is on is the COVID-19 inference. As mentioned in the original review – there was an expectation that seeing as this study data was collected during the pandemic, there would be some reflection on this? It is such an integral flash point in the understanding of the disparities in the global health space, and mental health itself was detrimentally affected. The paper currently doesn’t address the original comment/suggestion on how to integrate the contribution of COVID 19, but especially since the authors mention it – there must be some expansion and critique on this.**

**My original comment is as reference.**

**Additionally the authors now only mention that the data was collected in COVID in the last paragraph of the introduction, and they also mention only once in the results a comment by a participant.**

**If there is an intention to include a COVID reference then there must be some introductory thoughts around it, and more focus on its contribution in the discussion.**

Response R1.1: Thank you. We note that we alluded to COVID-19 without specifically interrogating it, as it was not our intended focus. To address your concerns, we now highlight the impact and implications of COVID-19 on mental health and the field of global mental health across several sections of the manuscript and hope that these revisions are adequate. In the Introduction, we clarified this as: “*In the case of mental health, this was made apparent during the COVID-19 pandemic. Pandemic consequences that affected mental health ranged from ruptures in day-to-day lives to destabilisation in international and national systems of trade, operations, and migration flows and exacerbation of widespread structural inequalities [**13-16**]. The World Health Organisation (WHO) highlighted interruptions to critical mental health services in 93% of countries, which jeopardised provision in an already underfunded field – especially in low and middle-income countries (LMICs) [**6-8**,* *17-19**]. With the pandemic perceived globally as a major economic and security threat, one effect was the heightened prioritisation of mental health as a global issue and increased attention to the field [**17-20**]. Whether this will last is unclear. It is therefore crucial to identify actors with the perceived authority and legitimacy to influence decision-making for the mental health field at the global level, as post-pandemic attention to mental health wanes. Existing attention needs to be properly coordinated towards reorganising mental healthcare systems and developing and implementing policies and interventions that improve psychosocial outcomes, so that this increased global prominence, even if temporary, has value [**21**].*” (p 3)

In the Findings section/Thematic Analysis sub-section, we expanded on this further across various instances: Firstly, we substantiated this as: “*Another shortcoming of such disconnection revealed itself during the COVID-19 pandemic, when it became apparent that some of the most powerful countries in Europe and North America struggled to contain SARS-Cov2 spread and address resulting mental health consequences. The ability of these countries to enact timely and appropriate policies to address urgent national needs and their corresponding global credibility was thus questioned. Previously regarded as models of mental health and policymaking, their influence may have weakened.*” (p 8)

Secondly, we substantiated further: “*While many participants acknowledged that the pandemic had detrimental and unequally distributed mental health consequences, it also increased global attention to the field. This propelled countries to come together with a greater sense of urgency and build upon existing efforts to constitute a more active response to shared mental health concerns across borders…”* (p 11)

Thirdly, we added a new quote and expanded on it as: “*The COVID-19 pandemic provided an opportunity to explain mental health in more relatable and comprehensible ways to political elites. Whilst it is crucial for them to address concerns about mental disorders and conditions, taking a more holistic view of mental health enables opportunities to integrate mental health support in the day-to-day lives of the public and should thus rank higher on agendas.”* (p 13)

In the Discussion section, we further examined COVID-19 impact and implications of by adding these paragraphs. Firstly, we clarified this as: “*The* COVID-19 *pandemic exposed the differential distribution of mental health outcomes amongst countries and populations and inadequacies in existing efforts to advance global mental health initiatives, demonstrating how mental health is continually affected by factors beyond health.*” (p 15)

Secondly: “*Notably, the COVID-19 pandemic demonstrated that worldviews and approaches exported from the Global North may not translate into effective mental health policies and interventions elsewhere, thereby contesting its epistemic authority…*” (p 16)

Thirdly: “*Several participants highlighted how COVID-19 provided an opportune moment for the global mental health field to further advocate for mental health to national governments and the wider global health community. COVID-19 made more tangible a subject that is stigmatised and often perceived to be abstract or irrelevant…*” (p 17)

Finally, we underscored the importance of taking action on mental health post-pandemic: “*Renewed attention to mental health and momentum amongst global health actors in response to the COVID-19 pandemic should be harnessed to further the prioritisation of mental health at the global level. Challenges in sustaining and translating this momentum into strategy and formalised action that are cognizant of the unique issues across geographic settings must also be addressed [**56**]. The future of mental health should therefore continue to encourage adoption of different perspectives, contextually attuned implementation, and reciprocal learning.*” (p 17 - 18)

**Reviewer 2 Comments**

**R2.1: First, thank you for the effort you have put into revising this article. It is much improved, with greater clarity, and addresses many of my initial concerns. However, there is still room for improvement. In both the introduction and abstract, the formulation of the research question could be made stronger and more explicit. A stronger research question would clearly connect the background presented to the relevance of the problem and the specific insights offered by the paper.**

Response R2.1: Thank you for your kind acknowledgment. To better clarify our research question in the abstract, we revised it as: “*We thus aimed to identify the most influential actors in global mental health and key challenges in the design and implementation of policies and interventions within this relatively nascent field, to provide suggestions on how mental health could be promoted and diversified at the global level to improve mental health outcomes.*” (p 2)

To better clarify the articulation of our research question in the Methodology, we substantiated it as: “*Who are the most influential individual and institutional actors in global mental health, what are key challenges in the design and implementation of global mental health policies and interventions, and how can the field be promoted and diversified within the broader global health context?*” (p 4)

**R2.2: Currently, the most immediate and relevant context for the problem is summarised as: "Against a backdrop of competing global health priorities, there is an urgent need to identify influential actors in this nascent field, to determine challenges, and to ascertain possible directions for its future development." While this identifies a need, the background of competing priorities is not explained in sufficient detail, nor is it clear why this creates a problem. Furthermore, the link between competing global health priorities and the need to identify influential actors is not adequately articulated. It is also unclear why identifying these actors will naturally lead to a determination of key challenges or inform future directions for the field’s development. Strengthening this argument will better support the research objectives and clarify their relevance to the global mental health field.**

Response R2.2: Thank you for your relevant suggestion. To address your point on the lack of clarity on competing priorities and why they pose a problem for the field of global mental health, we added: “*Despite this, other areas of global health continue to receive greater prioritisation and funding [**1**,* *2**]. While the neoliberal “deep core” of contemporary global health governance and policymaking frames global health issues as problems to be solved through cost-effective allocations of finite resources [**9**,* *10**], even relatively “unfettered healthcare markets” are heavily influenced by factors such as political interests, security concerns, or involvement of influential actors [**11**,* *12**]. Thus, global health fields that effectively advance a vision aligning with such factors (e.g. global health security) are generally prioritised [**3**,* *10**].”* (p 3)

To clarify the need to identify influential actors in global mental health, we added: “*With the pandemic perceived globally as a major economic and security threat, one effect was the heightened prioritisation of mental health as a global issue and increased attention to the field [**17-20**]. Whether this will last is unclear. It is therefore crucial to identify actors with the perceived authority and legitimacy to influence decision-making for the mental health field at the global level, as post-pandemic attention to mental health wanes. Existing attention needs to be properly coordinated towards reorganising mental healthcare systems and developing and implementing policies and interventions that improve psychosocial outcomes, so that this increased global prominence, even if temporary, has value [**21**]…*” (p 3)
